# Misattribution of Korea’s malaria elimination status in 1979

**DOI:** 10.4178/epih.e2025070

**Published:** 2025-12-10

**Authors:** Roma Seol, Youngtaek Kim

**Affiliations:** 1 Department of Preventive Medicine, Inje University College of Medicine, Busan, Korea; 2 Department of Preventive Medicine, Chungnam National University Hospital, Daejeon, Korea

**Keywords:** Malaria epidemiology, Misinformation, Information dissemination, World Health Organization certification

## Abstract

This study aimed to trace the origin and propagation of the common but incorrect belief that the World Health Organization (WHO) declared Republic of Korea (ROK) malaria-free in 1979. We conducted a source-based historical review of WHO *Weekly Epidemiological Record* (WER), regional reports from WHO’s Western Pacific Regional Office (WPRO), United States Department of Defense (DoD) health reports, and scholarly and web-based citations. WHO WER 1981 identified the Democratic People’s Republic of Korea (DPRK) as one of the countries that had eliminated malaria by 1979. WHO/WPRO, to which ROK belongs, reported that malaria had not been eliminated in ROK as of 1980. Misinterpretations within United States DoD documents incorrectly attributed this certification to ROK, resulting in widespread citation errors across academic literature and online sources. The misattribution of DPRK’s elimination status to ROK derives from a misreading of WHO records and has persisted for decades through repeated, unverified citations. Strengthening source accuracy and citation practices is essential for ensuring reliability in global health reporting.

Global malaria elimination efforts have frequently been shaped by milestone declarations issued by the World Health Organization (WHO), which function not only as scientific assessments but also as public health benchmarks. Among these statements, a widely repeated yet inaccurate claim asserts that WHO certified the Republic of Korea (ROK) as malaria-free in 1979. This assertion appears in government publications, academic articles, and online references, including Wikipedia. Our analysis, however, shows that the WHO certification applied instead to the Democratic People’s Republic of Korea (DPRK). The confusion was sustained through repeated misreadings and became more firmly entrenched following a 2009 publication by the United States Department of Defense (DoD).

This paper aimed to clarify the historical and documentary basis for this misattribution by examining primary WHO reports, Western Pacific Regional Office (WPRO) documentation, United States military health bulletins, and the subsequent dissemination of these sources in ROK and international academic literature. Notably, the absence of the DPRK from WPRO reporting after 1979, which reflects its non-member status in the regional office, created an interpretive void that contributed to later misunderstandings. Through this review, we sought to promote greater accuracy in epidemiological documentation and historical health interpretation.

Comparative analysis was conducted using WHO *Weekly Epidemiological Record* from 1979 to 1981, WPRO regional reports from 1980, and United States DoD (Health.mil) publications. Key phrases and citation patterns were traced through academic literature and publicly available websites from 1990 to 2023. Official ROK’s government documents were examined for any evidence indicating WHO certification of malaria elimination in the ROK.

WHO records explicitly identify DPRK as a country where malaria had been eliminated by 1979. In contrast, ROK was described as having reached low levels of incidence but had not achieved elimination (Table 1). A 2009 Health.mil article from the United States DoD mistakenly stated that WHO had declared ROK malaria-free in 1979, likely due to misinterpretation of the generic term “Korea” and incorrect reading of WHO data referring to DPRK (Table 2). This erroneous claim subsequently spread across Korean and international academic publications and became entrenched within online databases (Table 3).

WHO and WPRO documents from 1979 through 1980 consistently differentiate malaria elimination status in DPRK from the situation in ROK. Despite this clarity, a 2009 United States DoD publication misattributed DPRK’s elimination status to ROK. This mistake was later incorporated into ROK health publications and circulated widely through internet sources such as Wikipedia. Importantly, examination of Korea Disease Control and Prevention Agency archives and related reports reveals no evidence of any WHO declaration or certification indicating that ROK eliminated malaria.

Even prior to the United States DoD’s mistranslation in 2009, the reasons behind the spread of the inaccurate belief that the ROK was certified malaria-free by WHO in 1979 were difficult to trace. A domestic academic paper from 2000, likely the earliest known reference, stated that “it was declared eradicated in 1979 (WHO, 1981),” reflecting a misreading of data that referred to DPRK rather than ROK. Similar to the later misinterpretation by the United States DoD, this citation error has been repeated for years without correction.

However, the persistence of this misinformation until recently suggests that the authoritative reputation of the United States DoD may have substantially contributed to the ongoing acceptance and dissemination of the incorrect claim.

There is no evidence that WHO declared ROK malaria-free in 1979. WHO documentation confirms that only DPRK received malaria elimination status at that time. The misattribution appears to have originated from confusion within United States military communications and was later reinforced by unchecked academic and digital citations. ROK’s governmental agencies have never published any indication of WHO verification of malaria elimination for ROK. Correcting the historical record requires institutional transparency and more rigorous citation practices to prevent the continuation of such errors.

## Figures and Tables

**Table 1.** WHO and WPRO official recognition status: a comparison of primary sources

Source	Year	DPRK (North Korea)	ROK (South Korea)	Notes
WHO record [1]	1979	DPRK naturally malaria-free or have eradicated malaria.	Not mentioned in malaria-free list	DPRK implied via regional report wording
WHO/WPRO report [2]	1980	Not applicable (DPRK is not a WPRO member state)	“Low incidence in ROK; elimination not achieved”	WPRO evaluates only ROK, not DPRK

WHO, World Health Organization; WPRO, Western Pacific Regional Office; DPRK, Democratic People’s Republic of Korea; ROK, Republic of Korea.

**Table 2.** Source of misattribution: misreading of WHO data by United States DoD and others

Year	Source	Reported statement	Actual WHO position	Misattribution type
2007 [3]	Health.mil (DoD)	“WHO declared ROK malaria-free in 1979”	WHO never declared ROK malaria-free	DPRK misread as ROK
2010s [4]	USFK briefings, DoD derivatives	Korea declared malaria-free in late 1970s	DPRK only; ROK explicitly not eliminated in 1980 WPRO	Regional ambiguity and institutional repetition

WHO, World Health Organization; DoD, Department of Defense; DPRK, Democratic People’s Republic of Korea; ROK, Republic of Korea; WPRO, Western Pacific Regional Office; USFK, United States Forces Korea.

**Table 3.** Chronology of citation errors in academic and internet sources

Year	Source	Citation / Statement	Comment
2000	Ree HI, Korean J Parasitol [5]	“Malaria was declared eradicated in 1979 (WHO, 1981)”	WHO WER 1981, misreading
2003	Park JW et al., Am J Trop Med Hyg [6]	“ROK, was endemic on the Korean Peninsula for many centuries until the late 1970s, when the ROK was declared malaria free (WHO, 1981)”	WHO WER 1981, misreading
2009	Military Health System (USA DoD) [4]	WHO declared ROK malaria-free in 1979	Institutional misstatement
	Park JW et al., Korean J Parasitol [7]	“ROK was finally declared malaria-free in 1979”	WHO WER 1981, misreading
	Kim HC et al., Mil Med [8]	“Malaria was eradicated and the ROK declared “malaria free” in 1979”	WHO WER 1981, misreading
2013	Kim TS et al., Malar J [9]	...in 1979 when WHO declared the ROK to be malaria free”	WHO WER 1981, misreading
2018	Bahk YY et al., Korean J Parasitol [10]	In 1979, the WHO officially certified that Korea was a malaria-free country	No primary WHO source referenced
2010s	Wikipedia	ROK declared malaria-free in 1979	No WHO citation
2020s	Online blogs	Repetition of above claims	Copied from earlier errors

WHO, World Health Organization; WER, *Weekly Epidemiological Record*; ROK, Republic of Korea.
